# Brain imaging predictors and the international study to predict optimized treatment for depression: study protocol for a randomized controlled trial

**DOI:** 10.1186/1745-6215-14-224

**Published:** 2013-07-18

**Authors:** Stuart M Grieve, Mayuresh S Korgaonkar, Amit Etkin, Anthony Harris, Stephen H Koslow, Stephen Wisniewski, Alan F Schatzberg, Charles B Nemeroff, Evian Gordon, Leanne M Williams

**Affiliations:** 1 The Brain Dynamics Centre, University of Sydney Medical School - Westmead and Westmead Millennium Institute, Sydney, NSW 2145, Australia; 2 Brain Resource, Level 12, 235 Jones Street, Ultimo, Sydney, NSW 2007, Australia and Suite 200, 1000 Sansome Street, San Francisco, CA 94111, USA; 3 Sydney Medical School, University of Sydney, Camperdown, NSW 2050, Australia; 4 Department of Radiology, Royal Prince Alfred Hospital, Camperdown, NSW 2050, Australia; 5 Discipline of Psychiatry, University of Sydney Medical School: Western, Westmead Hospital, Sydney, NSW 2145, Australia; 6 Department of Psychiatry and Behavioral Sciences, Stanford University, 401 Quarry Road, Stanford, CA 94305, USA; 7 Sierra-Pacific Mental Illness Research, Education, and Clinical Center (MIRECC), Veterans Affairs Palo Alto Health Care System, 3801 Miranda Ave, Palo Alto, CA 94304, USA; 8 Department of Psychiatry and Behavioral Sciences, University of Miami Miller School of Medicine, Miami, FL 33136, USA; 9 BRAINnet Foundation, 71 Stephenson Street, Suite 400, San Francisco, CA 94105, USA

**Keywords:** Major depressive disorder, Antidepressant treatments, Imaging, Biomarker, iSPOT-D

## Abstract

**Background:**

Approximately 50% of patients with major depressive disorder (MDD) do not respond optimally to antidepressant treatments. Given this is a large proportion of the patient population, pretreatment tests that predict which patients will respond to which types of treatment could save time, money and patient burden. Brain imaging offers a means to identify treatment predictors that are grounded in the neurobiology of the treatment and the pathophysiology of MDD.

**Methods/Design:**

The international Study to Predict Optimized Treatment in Depression is a multi-center, parallel model, randomized clinical trial with an embedded imaging sub-study to identify such predictors. We focus on brain circuits implicated in major depressive disorder and its treatment. In the full trial, depressed participants are randomized to receive escitalopram, sertraline or venlafaxine-XR (open-label). They are assessed using standardized multiple clinical, cognitive-emotional behavioral, electroencephalographic and genetic measures at baseline and at eight weeks post-treatment. Overall, 2,016 depressed participants (18 to 65 years old) will enter the study, of whom a target of 10% will be recruited into the brain imaging sub-study (approximately 67 participants in each treatment arm) and 67 controls. The imaging sub-study is conducted at the University of Sydney and at Stanford University. Structural studies include high-resolution three-dimensional T1-weighted, diffusion tensor and T2/Proton Density scans. Functional studies include standardized functional magnetic resonance imaging (MRI) with three cognitive tasks (auditory oddball, a continuous performance task, and Go-NoGo) and two emotion tasks (unmasked conscious and masked non-conscious emotion processing tasks). After eight weeks of treatment, the functional MRI is repeated with the above tasks. We will establish the methods in the first 30 patients. Then we will identify predictors in the first half (n = 102), test the findings in the second half, and then extend the analyses to the total sample.

**Trial registration:**

International Study to Predict Optimized Treatment - in Depression (iSPOT-D). ClinicalTrials.gov, NCT00693849.

## Background

Major depressive disorder (MDD) is a leading cause of morbidity, mortality and disability for individuals aged 15 to 44 years [1]. A substantial percentage, (approximately 50%), of patients do not respond to antidepressant treatments and these individuals constitute a large proportion of the disease burden [2-5]. There is an urgent need for objective tests that can be obtained before initiating treatment to predict which patients will go on to show a clinical response to treatment, and to identify which type of treatment is best for the individual patient. Functional and structural brain imaging offers a means to identify treatment predictors, which are based upon the neurobiological mechanisms of these treatments, and the means to the pathophysiology of MDD and its subtypes.

The international Study to Predict Optimized Treatment for Depression (iSPOT-D) [6] is a large, international, multi-center, parallel model, clinical randomized trial which seeks to identify factors that predict treatment response to three common antidepressants. In iSPOT-D, we have combined a practical clinical trial design with the use of brain imaging to identify imaging predictors of treatment outcome in a real-world setting, along with other objective measures, such as electrophysiology and cognitive performance metrics. This paper presents the background, rationale and methodology for the imaging sub-study embedded within iSPOT-D.

Recent developments in neuroimaging have yielded powerful tools that enable quantification of both functional and static neural connectivity. These tools include diffusion tensor imaging (DTI), functional magnetic resonance imaging (fMRI), resting state fMRI, arterial spin labeling, positron emission tomography (PET) and high-resolution cortical mapping techniques that enable the automated, accurate localization of brain regions. Unfortunately, the majority of studies that have used these techniques to address treatment prediction in MDD have been underpowered, employing small cohorts (for example, n = 20 to 30), and they have typically focused upon a single imaging measure rather than seek convergence across multiple measures. Also, to date, no published study has been designed to test prediction across multiple antidepressants. However, a considerable body of data has examined the structural and functional differences that exist between the brains of depressed and non-depressed patients. These studies served to identify the brain circuits that represent viable targets for testing predictors of treatment outcome.

The rationale behind the use of three treatment arms in iSPOT-D is to identify what are the unique and common brain circuits that modulate response and remission to different types of anti-depressant medications, that is, selective serotonin reuptake inhibitors (SSRIs) versus selective norepinephrine reuptake inhibitors (SNRIs), or between the two different types of SSRIs. The receptor-level pharmacological actions of these medications may not be the best indicator of the mechanisms by which they lead to remission of depression, (which may take weeks, unlike receptor binding), and which is the primary focus of our analysis. For instance, sertraline and citalopram (the parent compounds for escitalopram) have been found to have differential effects in animals on hippocampal neuronal excitability [7], and are also known to differentially alter behavior in humans that are relevant to the brain circuits assessed in this study [8]. To date, there have been a few studies using small samples that examine the functional imaging predictors of response to individual antidepressants. These studies provide the basis for our rationale for the study. For example, using emotion activation tasks, depression has been associated with hyper-reactivity of amygdala circuits, which attenuates the following response to SSRIs [9-11]. The antidepressant sertraline has similarly been found to predict response and “normalize” amygdala hyperactivity and medial prefrontal hypo-activity, which characterize generalized social phobia, a disorder often comorbid with depression [12].

The need for a new mechanism-based approach to diagnosing and treating depression has been recognized in several initiatives. First, the research agenda for DSM-5 (the fifth edition of Diagnostic and Statistical Manual of Mental Disorders) emphasizes a need to translate neuroscience research findings into a new classification system for mental illnesses based on underlying brain circuitry [13]. Second, the Research Domain Criteria project - a National Institute of Mental Health initiative - aims to “develop, for research purposes, new ways of classifying mental disorders based on dimensions of observable behaviour and neurobiological measures” [14,15]. These objectives have also been touched upon by the recent Mayflower Action Group Initiative [16]. Imaging techniques, such as magnetic resonance imaging (MRI), provide a precise window into the circuits that play a role in the pathogenesis of depression and in the response to treatment, enabling the quantification of these circuits as potentially predictive “biomarkers”. The National Institutes of Health biomarker working group defined a biomarker as: “a characteristic that is objectively measured and evaluated as an indicator of normal biological processes, pathogenic processes, or pharmacologic responses to a therapeutic intervention” [17]. There are currently no clinically useful biomarkers for predicting response to antidepressant medications [18], a shortfall that the imaging sub-study of the iSPOT-D is designed to address.

In this paper, we characterize the key features of the iSPOT-D imaging sub-study. These features include (i) a focus on the discovery of imaging biomarkers that are likely to be clinically useful in the treatment of depression; (ii) a large and representative sample to achieve generalizable results, including a large percentage of treatment-naive participants; (iii) a translational design involving three common, current treatment regimes to provide an understanding of response heterogeneity; (iv) a highly standardized approach across sites to maximize the power of the data; and (v) a multi-modality MRI-based approach that provides convergent evidence that highlights key changes that are present.

Specifically, the iSPOT-D imaging study will serve to answer the following key questions:

1. What pre-treatment aspects of brain circuitry predict and moderate responses to anti-depressants?

2. What aspects of brain circuitry improve following treatment? We expect partially distinct circuitry to be involved in predicting response and remission to different antidepressants.

3. Do these predictive aspects of brain circuitry overlap with the core imaging features that are seen in depression when we compare depressed patients to healthy controls? Drawing on the available evidence to date, it is expected that those circuits impaired in depression will also be the mechanism by which antidepressants exert their mechanism of action. Thus, we will also assess if depression-related impairments are “normalized” post-treatment.

4. Are there additional distinctive alterations that identify specific “subtypes” of depression?

5. Are there associations between the imaging measures and the other data available from the iSPOT-D protocol?

## Methods/Design

### Study design

The structure of the parent iSPOT-D trial has been previously described in detail [6]. Briefly, it is a multi-center, international, prospective, parallel model, open-label randomized clinical trial involving 2,016 MDD patients aged 18 to 65 years. There are three treatment arms (escitalopram, sertraline and venlafaxine-XR) and a control arm, each with 672 participants. The healthy control participants are studied only at baseline, while the depressed participants are studied both at baseline and at eight weeks. The two sites for the imaging sub-study are the Brain Dynamics Center, University of Sydney (Sydney) and the Department of Psychiatry and Behavioral Sciences, Stanford University (Stanford). A target of 10% of iSPOT-D participants are to be recruited into the brain imaging sub-study with an overall target of approximately 200 participants with MDD (67 in each treatment arm) and 67 in the control group.

### Organizational structure/Acquisition sites

The iSPOT-D Executive Committee for the trial oversees the overall study. Supervision of the study on a day-by-day basis is performed at the Global Coordinating Center and Data Center (Sydney). Coordination of the study occurs via a Global Trial Coordinator and the executive management team. There are 20 global clinical sites involved in patient recruitment, though only two sites are involved in the imaging sub-study (Sydney and Stanford). The imaging-specific components of the sub-study are overseen by the senior imaging scientist (MSK) and the head of the imaging unit (SMG) at the central imaging site (University of Sydney, Westmead Hospital). The imaging data obtained at the secondary imaging site (Stanford University) are monitored at both the point of acquisition and the central imaging facility.

### Study participants

Details of the study inclusion–exclusion criteria are provided in Williams *et al.*[6]. In short, the primary diagnosis of nonpsychotic MDD is confirmed using the Mini-International Neuropsychiatric Interview (MINI) [2,3], according to DSM-IV criteria, and a score ≥16 on the 17-item Hamilton Rating Scale for Depression (HRSD-17) [4]. All MDD participants are either antidepressant medication (ADM) naïve or, if they had been previously prescribed an ADM, had undergone a wash-out period of at least five half-lives. Healthy control participants are extensively screened for the absence of Axis I disorders (using the MINI) and for an HRSD_17_ score <7.

**Figure 1:**
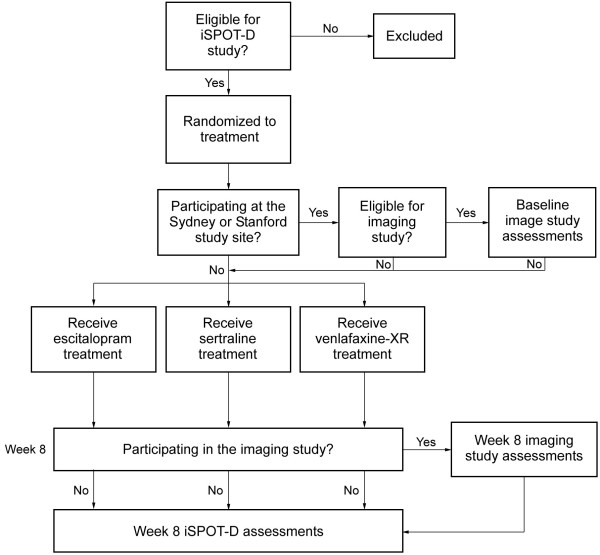
Trial flowchart.

Imaging participants are selected by sequentially recruiting patients at the time of enrollment (Figure 1). Additional MRI-specific exclusion criteria not applied to the general patients include the standard safety criteria for MRI scanning: a cardiac pacing/defibrillator device, claustrophobia, the presence of a magnet-incompatible metal prosthesis. Demographic information for both the MDD and control groups in the first 15% of participants is summarized in Table 1.

**Table 1:** Demographics for the MDD and control groups from the first 15% of participants

**Measures**	**Controls**	**MDD patients**
	**(n = 30)**	**(n = 30)**
Male/Females	12/18	12/18
Right-/Left-handed	26/4	26/4
	**Mean ± SD**	**Mean ± SD**
Age (years)	35.7 ± 14.1	41.2 ± 15.8
Education (years)	15.1 ± 2.8	14.5 ± 3.2
HRSD_17_	1.3 ± 1.4	19.2 ± 3.1^a^

^a^ significant difference with Control group at *P* <0.001, independent sample *t*-test.

HRSD_17_ 17-item Hamilton Rating Scale for Depression; MDD, Major depressive disorder.

#### Treatment

Participants were randomized to treatment using PhaseForward’s™ validated, Web-based Interactive Response Technology. A blocked randomization procedure was undertaken centrally (block size: 12, across sites). Neither participants nor investigators/raters were blinded to treatment assignment. The participant’s treating clinician (general practitioners and psychiatrists) prescribed ADMs adjusted dosage according to routine clinical practice.

### Ethical considerations

The study is being conducted according to the principles of the Declaration of Helsinki 2008 and the International Conference on Harmonization guidelines. Local regulations were also observed: The Australian Therapeutic Goods Association [19] and the United States Food and Drug Administration Code of Federal Regulations [20]. Prior to enrollment, the study procedures are fully explained to the study participant in accordance with the ethical guidelines of the institutional review board and written informed consent is obtained.

### MRI data acquisition

MRI studies are performed on a 3.0 Tesla GE SignaTwinspeedHDxtmagnet system (GE Healthcare, Milwaukee, WI, USA) utilizing an 8-channel (Westmead) or a 3.0 Tesla GE Discovery MR750magnet system utilizing a 32-channel (Stanford) phased-array head coil. Specialized equipment is also used to facilitate the fMRI data collection (Resonance Technology, Los Angeles, CA, USA) and the same standardized software is used for the delivery of functional stimulus (Stimsoft v1.2.1, Neuroscan, El Paso, TX). All imaging protocols were standardized and cross-site consistency established for data comparison. Table 2 shows cross-site validation of the T1 spoiled gradient recalled anatomical protocol using test-retest MRI scans collected on the same individual scanned at both the Sydney and Stanford magnetic resonance scanners. Table 3 presents a detailed summary of the acquisition parameters used. The imaging protocol takes approximately 1 hour and15 minutes to complete, including patient positioning. The protocol comprises both functional and structural MRI components. The full protocol is acquired at the initial visit; fMRI measures are replicated at the second time point only (imaging time: 45 minutes).

**Table 2:** Cross-site validation of the T1 SPGR anatomical protocol

**Intra-class correlation**	**Stanford scan 1**	**Stanford scan 2**	**Sydney scan 1**	**Sydney scan 2**
Stanford scan1	1.0			
Stanford scan 2	0.999	1.0		
Sydney scan1	0.996	0.996	1.0	
Sydney scan 2	0.997	0.996	0.996	1.0
Mean gray matter volume (mm^3^)	7,175 ± 5,527	7,198 ± 5,552	7,193 ± 5,620	7,226 ± 5,668

Intra-class reliability coefficients for volumetric data estimated for 68 brain gray matter regions using the T1 SPGR MRI protocol, using repeat scans done at the Stanford and Sydney site magnetic resonance scanners. Data show that inter-site scan reliability (for example, Stanford scan 1 - Sydney scan 1) is high and on the same comparative level as within-site scans (for example, Sydney scan 1 and Sydney scan 2). The mean (+ SD) for gray matter volume across the 68 brain regions for each scan is also listed.

*MRI* Magnetic resonance imaging, *SD* Standard deviation, *SPGR* Spoiled gradient recalled.

**Table 3:** Detailed summary of the MRI acquisition parameters

**Domain**	**Task/Scan type**	**MRI/Autonomic measures**	**Scan protocol/Task description**
Structural scans	T1	Gray/white matter volume	Scan protocol: 3D T1 weighted high resolution SPGR MRI scan, TR = 8.3 ms, TE = 3.2 ms, Flip angle = 11^0^, TI = 500 ms, NEX = 1, Matrix = 256 × 256, resolution = 1 mm × 1 mm, 180 contiguous 1 mm sagittal slices.
		Cortical thickness	
		Cortical surface area	
	Diffusion Tensor Imaging (DTI)	Fractional Anisotropy (FA), mean (MD), axial (AD), radial (RD) diffusivity measures for white matter regions and fiber tracts	Scan protocol: Spin echo DTI echo planar imaging scan, 42 directions, b value = 1,250, TR = 17,000 ms, TE = 95 ms, NEX = 1, Matrix = 128 × 128, resolution = 1.72 mm × 1.72 mm, 70 contiguous 2.5 mm axial/oblique slices covering the whole brain.
	PD/T2	Proton density	Scan protocol: 2D Fast Spin echo scan, TR = 4,000 ms, TE1 = 6.4 ms, TE2 = 102 ms, NEX = 1, Matrix = 256 × 256, resolution = 0.86 mm × 0.86 mm, ETL = 16, 70 contiguous 2.5 mm axial/oblique slices covering whole brain.
		T2 structural scan for clinical evaluation	
Functional scans	Oddball	Changes in fMRI BOLD signal	Scan protocol: Echo planar imaging scan, TR = 2,500 ms, TE = 27.5 ms, Flip angle = 90^0^, NEX = 1, Matrix = 64 × 64, resolution = 3.75 mm × 3.75 mm, 40 contiguous 3.5 mm axial/oblique slices covering whole brain in each volume, total 120 volumes.
		Average heart rate	
		Average skin conductance	Task description: 20 target (1,000 Hz) and 100 nontarget (50 Hz) tones presented one at a time at 75 db (50 ms each, ISI = 2.4 seconds).
	Continuous performance test (CPT)	Changes in fMRI BOLD signal	Scan protocol: See Oddball protocol
		Average heart rate	Task description: 120 stimuli are presented (B, C, D or G letters, for 200 ms each, ISI = 2.3 sec). Sixty were yellow letters to be held in working memory (no consecutive repetitions), 20 were 1-back sustained attention stimuli (consecutive repetitions of a letter in yellow) and 40 were perceptual baseline stimuli in white letters. Participants were measured for the number of errors or omissions.
		Average skin conductance	
	Go-NoGo	Changes in fMRI BOLD signal	Scan protocol: See Oddball protocol
		Average heart rate	Task description: 180 Go stimuli (word ‘press’ in green) and NoGo stimuli (word ‘press’ in red) each presented sequentially (500 ms each, ISI = .75 seconds). NoGo stimuli were not repeated more than three times in a row. Participants were measured for target detection rate, response time, errors of commission and errors of omission.
		Average skin conductance	
	Emotion (masked ‘nonconscious’; Then unmasked ‘conscious’)	Changes in fMRI BOLD signal	Scan protocol: See Oddball protocol
		Average heart rate	Task description: 240 3D evoked standardized facial expressions that depicted fear, anger, disgust, sadness, happiness or neutral were presented for 500 ms (ISI = .75 seconds). Stimuli were grouped into eight faces expressing the same emotion and were repeated five times in a pseudorandom order. Participants were assessed for explicit emotion processing for the ‘Masked’ condition.
		Average skin conductance	The same set of facial expressions was presented for the ‘Unmasked’ condition; however, backward masking (expression presented for 10 ms immediately followed by a neutral face for 190 ms) was used to prevent conscious awareness of facial expression.
Additional scan	Phase map	Map magnetic field in homogeneities for EPI distortion correction	Scan protocol: Dual echo MRI scan, TE1 = 10 ms, TE2 = 12.5 ms, TR = 700 ms, Flip angle = 15^0^, NEX = 1, Matrix = 128 × 128, 40 contiguous 3.5 mm axial/oblique slices.

*2D* Two-Dimensional, *3D* Three-Dimensional, *BOLD* Blood oxygen level dependent; *EPI*, Echo-planar imaging, *ETL* Echo-train length, *fMRI* Functional magnetic resonance imaging, *ISI* Inter-stimulus interval, *MRI* Magnetic resonance imaging, *NEX* Number of excitations, *PD* Proton density, *SPGR* Spoiled gradient recalled, *TE* Echo time, *TR* Repetition time.

The structural dataset comprises three scans: a high-resolution three-dimensional (3D) T1-weighted volume dataset (1 mm isotropic resolution; repetition time (TR) 8.3 ms; echo time (TE) 3.2 ms; inversion time (TI) 500 ms), DTI data (1.7 × 1.7 in-plane resolution; 2.5 mm slice thickness; TR 17,000 ms; TE 95 ms) and dual echo data (0.86 × 0.86 in-plane resolution; 2.5 mm slice thickness; TR 4,000 ms; TE1 6.5 ms; TE2 102 ms).

### fMRI protocol

The functional dataset involves three cognitive tasks and two emotion tasks that assess cognitive thinking and emotion processes. The methods have been fully described elsewhere but are summarized below for completeness [21,22].

The cognitive tasks include an auditory oddball task, a continuous performance task and the Go-NoGo task. The oddball task is used for evaluating attention modulation. The stimuli consist of 20 high-pitched (1,000 Hz at 75 dB) ‘target’ tones and 100 lower-pitched (500 Hz at 75 dB) ‘non-target’ tones. In the continuous performance task, participants view a series of 120 letters (B, C, D or G) and are instructed to press a button when the same yellow letter appears twice in a row (a ‘1-back’ design). To provide a baseline, 40 stimuli are presented in white, which participants are instructed to ignore. The Go-NoGo task is used to assess impulsivity (automatically-generated ‘Go’ responses) versus inhibition (‘NoGo’ responses). ‘Go’ responses are defined by the word ‘press’ presented in GREEN, while ‘NoGo’ responses are defined by the word ‘press’ presented in RED.

The emotion tasks consist of an unmasked conscious emotion processing task and a masked non-conscious emotion processing task. The unmasked emotion processing task [23,24] was developed from a standardized set of 3D evoked facial expressions that depict fear, anger, disgust, sadness, happiness or are neutral [25]. There are a total of 240 stimuli grouped in blocks of eight faces of the same emotion, with each emotion block repeated five times. In the masked non-conscious emotion processing task, the same set of facial emotion stimuli are used but each stimulus consists of the emotion face presented non-consciously (for 10 ms), followed immediately by a neutral face mask stimulus (for 150 ms). This paired presentation is designed to prevent conscious awareness of the emotion stimulus [23,24].

### Data processing pipeline

Data are processed using a custom-built automated pipeline run via scripts in the MATLAB 7.5 software (MathWorks, Natick, MA, USA). Processing tasks are performed by a cluster of 18 custom-built computer nodes running Linux (CENTOS 5.0 distribution); tasks are assigned, queued and coordinated using a central computer. Specific details of the analysis procedures are provided below. In summary, the raw DICOM data from the magnetic resonance scanner are imported using a script and converted to NIfTI format, then is run through sequential routines drawn from the Functional MRI of the Brain (FMRIB) Software Library (FSL) release 4.1.3 (http://www.fmrib.ox.ac.uk/fsl) [26], the Freesurfer image analysis suite (version 4.3) (documented and freely available for download online (http://surfer.nmr.mgh.harvard.edu/) [27], SPM8 software [28] and custom-written routines that have been described in previous publications [29-32]. Anonymized data are transferred between the two imaging sites via a secure ftp connection.

### Data storage

Anonymized data are kept in a structured file system that includes selected intermediate steps to permit the easy recalculation of data for specific analyses. Off-site back-ups of data are kept in a secure facility.

### Specific MRI analyses

#### Volumetric and cortical thickness data

Measurements of cortical thickness and volume are made as described previously [33] using Freesurfer. Briefly, cortical surface reconstruction and volumetric segmentation are performed in an automated manner on the 3D T1 weighted structural images using the Freesurfer image analysis suite (version 4.3). The full technical details of these procedures are described elsewhere [34-45]. All cortical segmentation and labels are manually inspected for accuracy. Voxel-based morphometry (VBM8) analysis as implemented in SPM8 is also performed.

#### DTI processing - tract-based spatial statistical (TBSS) and tractography analysis

We have previously presented the TBSS-based analysis used to process our DTI data [46]. DTI data are preprocessed and analyzed using the Oxford Centre for FMRIB Diffusion Toolbox and TBSS software tools, part of the FSL release 4.1.3 (http://www.fmrib.ox.ac.uk/fsl) [26,47,48]. The raw DTI data for each participant are first corrected for head movement and eddy current distortions. Diffusion tensor models are then fitted independently for each voxel within the brain mask and images of fractional anisotropy (FA), mean diffusivity (MD) and first (λ1), second (λ2) and third Eigen value (λ3) are generated for each participant. FA images from each participant are then aligned to the FMRIB58_FA template and transformed into Montreal Neurological Institute (MNI) 152 1 mm^3^ standard space using FMRIB’s nonlinear registration tool FNIRT [49,50]. Next, an average FA image is generated and thinned to create a white matter skeleton representing the centers of all white matter tracts common to all participants. This FA skeleton is then thresholded to FA ≥0.3 to include the major white matter pathways but avoid peripheral tracts that are more vulnerable to inter-participant variability and/or partial volume effects with gray matter. Each participant’s aligned FA image is then projected onto the mean FA skeleton. These skeletonized participant data will be used for performing voxel-wise cross-participant statistics using permutation testing procedures. Clusters with significant differences will be labeled using the Johns Hopkins University International Consortium for Brain Mapping (JHU ICBM)-DTI-81 white matter labels atlas [51]. DTI tractography will also be performed using a multi-fiber diffusion probabilistic model [52]. This method estimates probability distributions for one or more fiber populations at each brain voxel. DTI tractography will allow obtaining a more precise quantification of the exact orientation and trajectory of WM fiber tracts.

#### Functional MRI data analysis

The fMRI analysis procedures are described in detail elsewhere [22]. In brief, pre-processing and analysis are performed using SPM8 software (http://www.fil.ion.ucl.ac.uk/spm), and involve motion correction, global signal estimation and removal, estimation of normalization transforms from functional to standard MNI space, spatial smoothing, and then application of a high-pass filter. A canonical hemodynamic response function (HRF)-convolved event-related model is used to model the blood oxygen level dependent (BOLD) responses for the three cognitive tasks. An HRF-convolved box-car function is used to model the BOLD response for both of the emotional processing tasks. Contrast images for each cognitive thinking and emotion processing task are generated and will be evaluated for voxel-wise comparisons in BOLD signal for the whole brain. A significance threshold of *P* <0.05 corrected for multiple comparisons will be used. Clusters with significant differences will be labeled using the Talairach Atlas.

#### Region of interest definitions

To test specific hypotheses of regional involvement in volume measurements, fMRI activation tasks and, in the resting state-fMRI, standardized regions of interest (ROI) have been defined using the WFU PickAtlas software as a basis [53]. For selected ROIs without standardized available masks, custom definitions will be generated using the automated coordinate extraction method within the NeuroSynth framework (http://neurosynth.org) [54]. This environment uses data derived from a machine-learning technique (naive Bayes classification) to estimate meta-analysis maps of activation maps based on specified search terms. For DTI data, the JHU white matter atlas will be used with the TBSS processed data to define white matter fiber bundles. DTI tractography will also be performed to define individualized fiber tracts for each participant.

### Quality control

MRI data are evaluated at the time of acquisition by the radiographer. Each MRI study (excluding the fMRI data) is reported by a radiologist at the site of acquisition (Departments of Radiology, Westmead Hospital and Stanford University) to exclude structural abnormalities. The imported NIfTI files are visually inspected by the local imaging scientist prior to analysis. MRI protocols were standardized by using careful synchronization of parameters between sites, scanning of a human phantom on multiple occasions (MSK, SMG), inter-site visits and ongoing clinical assessment (see Table 2). Routine quality control of the analysis pipeline was performed using standardized outputs generated to reflect the quality of motion correction, image registration and parameter maps for the functional data, fractional anisotropy images for the DTI data and cortical-subcortical parcellations from Freesurfer for the structural T1 data.

### Analytic approach

Analyses of iSPOT-D imaging data will be conducted in three phases.

#### Phase 1 - identification of putative imaging biomarkers for MDD and subtype classification

Phase 1 includes the first 15% of the MDD cohort tested at the Sydney site (that is, 30 MDD participants, 30 matched controls). Analyses will test for the convergence of structural and functional imaging data for brain circuitry dysfunction in depression (that is, Question 1). These analyses will replicate existing analyses in the literature using structural and functional imaging in MDD. We provide outcomes from these Phase 1 analyses in this report.

#### Phase 2 - testing of biomarkers, hypothesis generation for testing against Phase 3 data

Phase 2 includes the first 50% of the MDD cohort tested at the Sydney site (that is, 102 MDD participants with 34 in each of the three treatment arms, and 34 matched controls). Analyses will mainly identify baseline brain circuitry predictors of treatment response, core brain circuitry associated with depression and its subtypes, and brain circuits that change with treatment (that is, Questions 1 through 4).

#### Phase 3 - replication of early findings, testing associations in pooled data

Phase 3 includes the full MDD cohort tested at the Sydney site (204 MDD participants, 68 matched controls) and Stanford site (51 MDD participants, 17 matched controls). Analyses will replicate findings from Phase 2 analyses using the entire Sydney cohort and, a separate replication will be performed in an independent Stanford cohort. The issue of inter-site variance between the two imaging sites will be addressed prior to pooling data. We will first check for systematic differences, if these exist then they will be modeled out from the analysis, using site as a covariate. The full cohort will also provide sufficient power to test for associations of imaging measures with other data available from the iSPOT-D study protocol (Question 5).

As a first pass, each of the MRI measures will be analyzed independently and then combined into a multimodal multiple regression analysis.

### Power Calculation/Effect size

For analysis of predictive measures (for example, imaging measures that predict treatment response or remission due to a specific agent, or predict treatment resistance assuming that about 50% of participants will respond [55]) at a statistical power of 80% and an effect size of 1 standard deviation, the sample size required is 17, (that is, assuming each treatment has N of approximately 102/3 -- > N = 34; meaning n(R) = 34*.5 = 17 responders/non-responders). This permits us to analyze single treatment arms at the 50% data level, enabling replication of effects with the second 50% of data.

### Future exploratory analyses

The high-powered imaging dataset available from the iSPOT-D study will also provide the opportunity for new analyses on the cutting edge of the imaging field. Below, we list some of the novel analyses that we plan to conduct using the imaging data.

#### Whole brain mapping of inter-regional connectivity using DTI

We quantify inter-regional white matter connectivity by defining cortical ROIs based on the cortical thickness parcellation, then determining the number of fibers connecting each region. Fiber tracking utilizes a multi-fiber diffusion probabilistic model that estimates probability distributions for one or more fiber populations at each brain voxel [52]. Tractography is performed by sequentially using each of the cortical labels as seed and the remaining labels as targets. The resulting dataset, a 70 × 70 inter-regional connectivity matrix for each participant, will then be subjected to either a pattern-based analysis (for example, principle components analysis [56] or group theory [57-60]), or a focused, hypothesis-driven analysis of specific inter-regional connectivity. This method has been previously described using Phase 1 data from the first 15% of the iSPOT-D dataset [56]. We used a pattern analysis approach to analyze these data to show a 92% accuracy in characterizing the MDD and control cohorts.

#### White matter hyperintensity (WMH) analysis

WMH will be measured using a custom, semi-automated routine using dual-echo data (proton density and spin echo data) combined with reformatted T1W data in the axial plane. Initial lesions are selected using OsiriX (http://www.osirix-viewer.com) [61]; then lesions are quantified in MATLAB using a multi-modality (T1, T2, Proton Density) cluster analysis technique (details to be published in a future paper). A separate qualitative analysis will also be performed using the well-validated Schelton’s score [62].

#### Volume and connectivity analysis of the basal ganglia, amygdala, thalamus, brainstem and cerebellum

Several novel analytical approaches currently underway are designed to obtain more detailed connectivity information from the deep gray structures and from the cerebellum and brainstem structures, along the lines of previous probabilistic approaches [47].

#### Resting state fMRI analysis

The fMRI task data will be used to extract resting state fMRI data. To identify resting state connectivity, the task effects are modeled within a general linear model framework. These effects are removed and the remaining (residual) time-series signal is analyzed using SPM8 software. This method of obtaining resting connectivity from task fMRI data has been previously established and validated against non-task-derived resting state data [63,64].

#### Small world analysis of brain networks

The application of graph theory methods to imaging data has been useful in extracting sensitive measures of brain connectivity [57-60]. We will test how these measures are altered in MDD and test their prognostic value.

## Results

Here we present a summary of outcomes to date from the analysis of Phase 1 using multimodality data (DTI, fMRI and T1). Using these measures, we test for convergent evidence of core brain circuitry dysfunction in depression, looking at the baseline data for the first 15% of subjects (prior to treatment). For this analysis, we investigate the mechanisms of this circuitry, with a specific focus on the dorsolateral prefrontal cortex, anterior cingulate cortex (ACC) and amygdala.

Our preliminary data (first 15% of subjects) demonstrate prefrontal dysfunction in MDD, with contributions from DTI, fMRI and T1 datasets. In the melancholic MDD subgroup, we have shown alterations of white matter within the white matter fiber tracts that are associated with the prefrontal, limbic and thalamic circuitry [46,56]. For the same cohort, we also found prefrontal dysfunction using fMRI data from all five cognitive and emotion processing tasks: MDD participants were distinguished by a distinctive biosignature of hypoactivation of the dorsolateral prefrontal cortex during working memory updating and during conscious negative emotion processing, hyperactivation of the dorsomedial prefrontal cortex (ACC) during working memory and response inhibition cognitive tasks, and hypoactivation of the dorsomedial prefrontal cortex (ACC) during conscious processing of positive emotion tasks [22].

**Figure 2:**
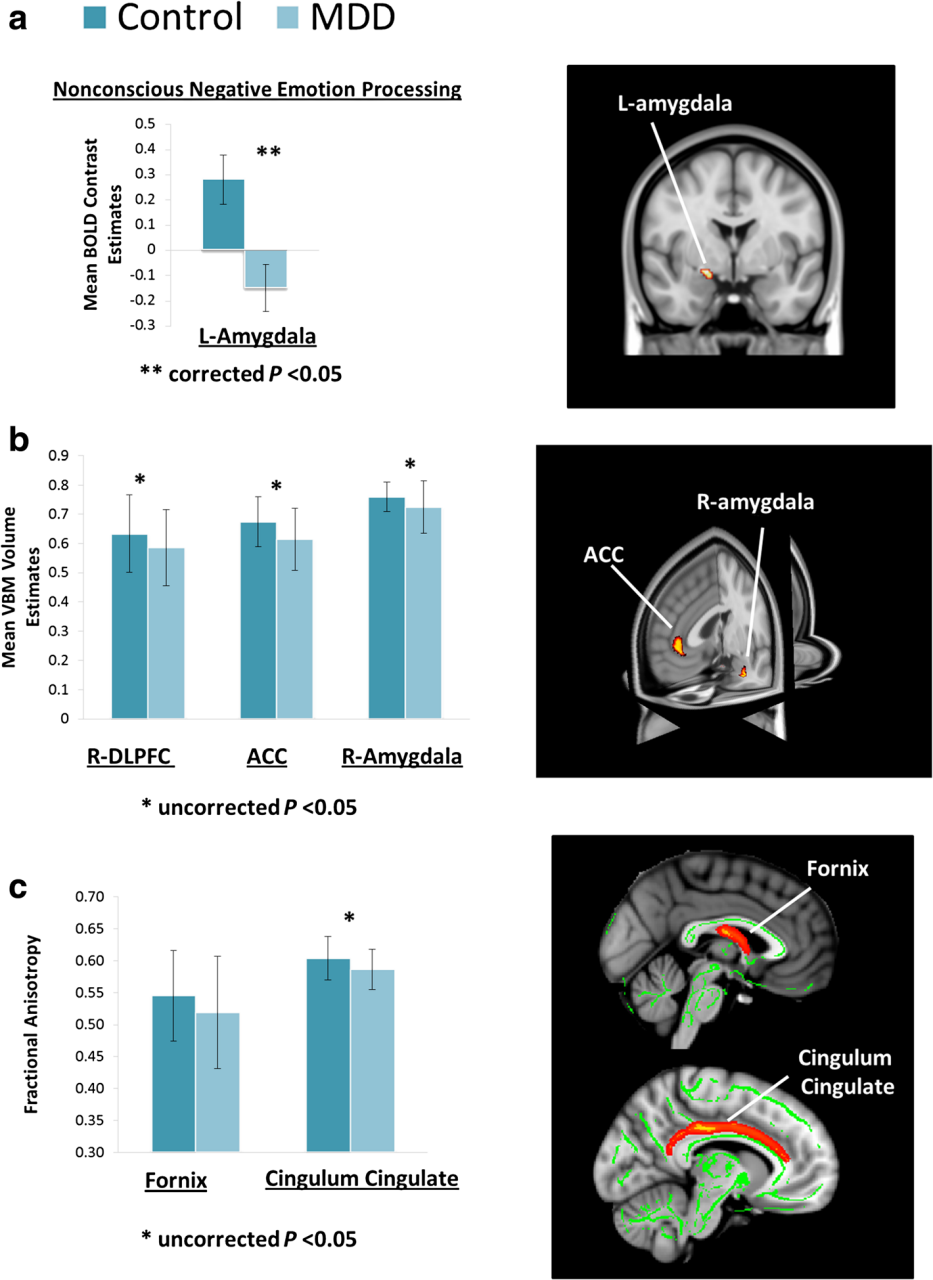
**Summary of preliminary functional MRI data from the first 15% of participants (MDD versus controls). (a)** A coronal view (right) demonstrating significant hypoactivation of the left amygdala for non-conscious negative emotion processing. Comparison between the MDD and control groups (left) shows a significant difference (corrected *P* <0.05). **(b)** Significant reductions in gray matter volume (uncorrected *P* <0.05) are shown in the right amygdala, right dorsolateral prefrontal cortex and anterior cingulate regions. **(c)** FA in the fornix and cingulum portion of the cingulate bundle was significantly lower (*P* <0.05) in the MDD group in comparison to controls.

Further data from the first 15% of subjects highlight the central role of the limbic regions and the ACC in MDD using convergent evidence from fMRI, structural T1 and DTI data. The fMRI data and DTI data were analyzed using the methods described in Korgaonkar *et al.*[22,46], while the structural T1 weighted data were analyzed using the methods described above. We tested for MDD-Control group differences in the fMRI BOLD signal and gray matter volume in the ACC and amygdala, as well as for FA differences in two white matter fiber bundles associated with these regions, that is, the cingulum cingulate bundle and fornix respectively. A significant difference (corrected *P* <0.05) between the MDD and control group was found in the left amygdala for non-conscious negative emotion processing, with hypoactivation of this region seen in the MDD group (Figure 2a) (significant differences in ACC and DLPFC are already reported in [22]). A reduction in gray matter volume (uncorrected *P* <0.05) was also seen in both the amygdala and ACC regions (Figure 2b). FA in the cingulum cingulate bundle was also found to be significantly reduced (*P* <0.05) in the MDD group in comparison to controls (Figure 2c).

## Discussion

There are currently no clinically useful predictors of MDD response or remission with the antidepressants escitalopram, sertraline or venlafaxine-XR. MRI measures of brain circuitry and function hold great potential as “biomarkers” in psychiatric disease; however, there is a lack of data regarding their use in predicting treatment response in MDD. The imaging sub-study of iSPOT-D seeks to provide high-level evidence regarding which aspects of brain circuitry predict and moderate response to these antidepressants.

In this paper, we have outlined the protocol and rationale for the imaging sub-study of iSPOT-D. We summarized the already-published outcomes from the first 15% of the data and also presented some additional supportive data. These multimodality outcomes provide the first convergent evidence of dysregulation in both the activation and connectivity of the prefrontal-limbic circuitry in depression. Analysis of the 50% data will focus on predictive measures of treatment response, with an *a priori* focus on these circuits. The careful design of our study, its power and the focus on multimodality data maximize the possibility of achieving our aim of generating imaging-based metrics that have clinically relevant predictive value.

At the completion of iSPOT-D and the imaging sub-study delineated here, we will investigate how imaging measures of brain circuitry relate to other clinical, genetic, behavioral and physiological units of analysis in order to test combined predictors of treatment outcome.

## Trial status

Enrollment is ongoing. Final data collection date for primary outcome measure is estimated to be August 2013. Study completion date is estimated to be December 2013.

## Abbreviations

2D: Two-dimensional; 3D: Three-dimensional; ACC: Interior cingulate cortex; ADM: Antidepressant medication; BOLD: Blood oxygen level dependent; DSM-5: the fifth edition of the Diagnostic and Statistical Manual of Mental Disorders; DTI: Diffusion tensor imaging; EPI: Echo-planar imaging; ETL: Echo-train length; FA: Fractional anisotropy; fMRI: Functional magnetic resonance imaging; FMRIB: Functional MRI of the brain; FSL: Functional MRI of the brain software library; HRF: Hemodynamic response function; HRSD17: 17-item Hamilton Rating Scale for Depression; ISI: Inter-stimulus interval; iSPOT-D: International study to protect optimized treatment for depression; JHU ICBM: Johns Hopkins University International Consortium for Brain Mapping; MD: Mean diffusivity; MDD: Major depressive disorder; MINI: Mini-international neuropsychiatric interview; MNI: Montreal neurological institute; MRI: Magnetic resonance imaging; NEX: Number of excitations; PET: Positron emission tomography; ROI: Regions of interest; SD: Standard deviation; SNRIs: Selective norepinephrine reuptake inhibitors; SPGR: Spoiled gradient recalled; SSRIs: Selective serotonin reuptake inhibitors; TBSS: Tract-based spatial statistical; TE: Echo time; TR: Repetition time; VBM8: Voxel-based morphometry; WMH: White matter hyperintensity; λ1: First Eigen value; λ2: Second Eigen value; λ3: Third Eigen value.

## Competing interests

SMG has received consulting fees from the Brain Resource Company. AH has received consultancy fees from Eli Lilly, Janssen-Cilag and Lundbeck Australia. He has received payments for educational sessions run for Astra Zeneca, Janssen Cilag and Eli Lilly. He has run educational sessions Reed Business Information. He is an investigator on industry sponsored trials by Hoffman-La Roche, Janssen-Cilag Australia and Brain Resource Company. SHK serves as a consultant and has stock options with Brain Resource Company. SW has received fees as a consultant from CyberonicInc, ImaRx Therapeutics Inc, Bristol-Myers Squibb Company, Organon, Case-Western University and Singapore Clinical Research Institute. AFS has received fees as a consultant from Brain Cells, CeNeRxm, CNS Response, Corcept, Glaxo-Smith Kline, Merck and Company, Neuronectics, PharmaNeuroBoost, Sanofi-Aventis and Takeda. He is a stock holder in Amnestix, Brain Cells, CeNeRx, Corpect, Forest, Merck and Company, Neurocrine, Pfizer, PharmaNeuroBoost, Somaxon and Synosis.

CBN has received a Scientific Advisory Board/Board of Directors fee from AstraZeneca, PharmaNeuroboost, Forest Laboratories, NARSAD, Quintiles, Janssen/Ortho-McNeil, Mt. Cook PharmaInc, George West Mental Health Foundation and NovaDelPharma. He holds stock in Corcept, CeNeRx, ReVax, PharmaNeuroboost and NovadelPharma. CBN has patents for methods and devices for the transdermal delivery of lithium (US 6,375,990 BI) and a method to estimate serotonin and norepinephrine transporter occupancy after drug treatment using patient or animal serum (provisional filing April, 2001). EG is founder of Brain Resource Ltd and receives income as its Chief Executive Officer and Chairman. He has stock options in Brain Resource Ltd. LMW has received consulting fees and stock options in Brain Resource Ltd, and is a stock holder in Brain Resource Ltd. She has received Advisory Board fees from Pfizer.

MSK and AE have no competing interests to disclose.

## Authors’ contributions

SMG made substantial contributions to conception and design of the trial, provided important intellectual content to the manuscript, and was substantially involved in drafting and revising the manuscript. MSK, AE, AH, SHK, SW, AS, CBN and EG made substantial contributions to the conception of the trial, and were substantially involved in drafting and revising the manuscript. LMW made substantial contributions to the conception and design of the trial, provided important intellectual content to the manuscript, and was substantially involved in drafting and revising the manuscript. All authors gave final approval of the submitted version of the manuscript.

## Authors’ information

SMG is an Associate Professor at the Sydney Medical School in the University of Sydney. He designed the imaging protocol for the study and with MSK has led the analysis and processing of the imaging data. He is a clinician-scientist with a strong interest in translational imaging. He completed his specialty training in radiology at Royal Prince Alfred Hospital and his doctorate in MRI imaging of the brain at Oxford University. His clinical and research interests are focused on cardiac and neuroimaging. He is currently a Sydney Medical Foundation Fellow at the University of Sydney and Head of Imaging at the Brain Dynamics Center.

MSK is a Senior Imaging Scientist on the study. He completed his Masters in Medical Physics and doctoral degree in MR Imaging at the department of Biomedical Engineering at Stony Brook University, NY. He is currently appointed as Senior Imaging Scientist and Director of Brain Dynamics Center, Sydney Medical School - Westmead.

AE is Assistant Professor of Psychiatry and Behavioral Sciences at Stanford University, an Investigator in the Sierra-Pacific Mental Illness Research Education and Clinical Center (MIRECC) at the Veterans Affairs Palo Alto Health Care System, and a board-certified psychiatrist. He directs a laboratory focused on understanding the neurobiology of mood and anxiety disorders.

AH is an Associate Professor in the Discipline of Psychiatry at the University of Sydney and is the Clinical Director at the Brain Dynamics Centre, Westmead Millennium Institute at the University of Sydney. He is a senior staff specialist for the Western Sydney Local Health Network. His interests have centered upon the treatment, psychophysiology and neuroimaging of young people with psychosis and depression.

SHK is a senior research fellow in the Miami University School of Psychiatry and Behavioral Sciences and a Director on the board of the BRAINnet Foundation. He recently served as the Research Director for the American Foundation for Suicide Prevention. He was the first Director of the Neuroscience Research Branch at the NIMH where he was responsible for initiating new research programs, including Human Brain Imaging and the Human Brain Project. SHK was founder of the International Neuroinformatics Coordinating Facility. He currently serves as a consultant to Brain Resources.

SW is a professor in the Department of Epidemiology and Co-Director of the Epidemiology Data Center, University of Pittsburgh. His secondary appointment is in psychiatry. He is an Associate Dean for Research in the Graduate School of Public Health.

AFS is the Kenneth T. Norris, Jr. Professor in Psychiatry and Behavioral Sciences at Stanford University. Before this, he built the department of Psychiatry and Behavioral Sciences as chair of the department.

CBN is the Leonard M. Miller Professor and Chairman of the Department of Psychiatry and Behavioral Sciences, University of Miami.

EG is the founder and Chairman of Brain Resource, which has established the first standardized battery for assessing human behavior and brain function and structure.

LMW is acting Professor in Psychiatry and Behavioral Sciences at Stanford University, and holds a joint appointment as Professor in Cognitive Neuropsychiatry at Sydney Medical School.

## References

[B1] Mathers C Fat DM Boerma JT The Global Burden of Disease: 2004 update 2008 Geneva: World Health Organization

[B2] Exner C Lange C Irle E Impaired implicit learning and reduced pre-supplementary motor cortex size in early-onset major depression with melancholic features J Affect Disord 2009 119 156 162 10.1016/j.jad.2009.03.015 19345999

[B3] Shah PJ Glabus MF Goodwin GM Ebmeier KP Chronic, treatment-resistant depression and right fronto-striatal atrophy Br J Psychiatry 2002 180 434 440 10.1192/bjp.180.5.434 11983641

[B4] Soriano-Mas C Hernández-Ribas R Pujol J Urretavizcaya M Deus J Harrison BJ Ortiz H López-Solà M Menchón JM Cardoner N Cross-sectional and longitudinal assessment of structural brain alterations in melancholic depression Biol Psychiatry 2011 69 318 325 10.1016/j.biopsych.2010.07.029 20875637

[B5] Greenberg P Corey-Lisle PK Birnbaum H Marynchenko M Claxton A Economic implications of treatment-resistant depression among employees Pharmacoeconomics 2004 22 363 373 10.2165/00019053-200422060-00003 15099122

[B6] Williams LM Rush AJ Koslow SH Wisniewski SR Cooper NJ Nemeroff CB Schatzberg AF Gordon E International study to predict optimized treatment for depression (iSPOT-D), a randomized clinical trial: rationale and protocol Trials 2011 12 4 10.1186/1745-6215-12-4 21208417 PMC3036635

[B7] Igelstrom KM Heyward PM Inhibition of hippocampal excitability by citalopram Epilepsia 2012 53 2034 2042 10.1111/j.1528-1167.2012.03660.x 22946760

[B8] Riedel WJ Eikmans K Heldens A Schmitt JA Specific serotonergic reuptake inhibition impairs vigilance performance acutely and after subchronic treatment J Psychopharmacol 2005 19 12 20 10.1177/0269881105048887 15671124

[B9] Arnone D McKie S Elliott R Thomas EJ Downey D Juhasz G Williams SR Deakin JF Anderson IM Increased amygdala responses to sad but not fearful faces in major depression: relation to mood state and pharmacological treatment Am J Psychiatry 2012 169 841 850 10.1176/appi.ajp.2012.11121774 22854930

[B10] Ruhe HG Booij J van Weert HC Reitsma JB Franssen EJ Michel MC Schene A [Dose-escalation of SSRIS in major depressive disorder. Should not be recommended in current guidelines] Tijdschr Psychiatr 2010 52 615 625 20862644

[B11] Victor TA Furey ML Fromm SJ Bellgowan PS Ohman A Drevets WC The extended functional neuroanatomy of emotional processing biases for masked faces in major depressive disorder PLoS One 2012 7 e46439 10.1371/journal.pone.0046439 23056309 PMC3466291

[B12] Phan KL Coccaro EF Angstadt M Kreger KJ Mayberg HS Liberzon I Stein MB Corticolimbic brain reactivity to social signals of threat before and after sertraline treatment in generalized social phobia Biol Psychiatry 2013 73 329 336 10.1016/j.biopsych.2012.10.003 23164370 PMC3557668

[B13] Charney D Barlow D Botteron K Cohen J Kupfer DJ, First MB, Regier DA Neuroscience Research Agenda to Guide Development of a Pathophysiologically Based Classification System A research agenda for DSM-V 2002 American Psychiatric Publishing, Incorporated 31 84

[B14] National Institute of Mental Health The National Institute of Mental Health Strategic Plan 2008 Bethesda, MD: National Institute of Mental Health. NIH publication 08–6368

[B15] Insel T Cuthbert B Garvey M Research Domain Criteria (RDoC): toward a new classification framework for research on mental disorders Am J Psychiatry 2010 167 748 751 10.1176/appi.ajp.2010.09091379 20595427

[B16] Koslow SH Williams LM Gordon E Personalized medicine for the brain: a call for action Mol Psychiatry 2010 15 229 230 10.1038/mp.2009.147 20065956

[B17] Atkinson AJ Colburn WA DeGruttola VG DeMets DL Downing GJ Hoth DF Oates JA Peck CC Schooley RT Spilker BA Woodcock J Zeger SL Biomarkers and surrogate endpoints: preferred definitions and conceptual framework Clin Pharmacol Ther 2001 69 89 95 11240971 10.1067/mcp.2001.113989

[B18] Rush AJ Fava M Wisniewski SR Lavori PW Trivedi MH Sackeim H Thase ME Nierenberg A Quitkin FM Kashner TM Kupfer DJ Rosenbaum JF Alpert J Stewart JW McGrath PJ Biggs MM Shores-Wilson K Lebowitz BD Ritz L Niederehe G STAR*D Investigators Group Sequenced treatment alternatives to relieve depression (STAR*D): rationale and design Control Clin Trials 2004 25 119 142 10.1016/S0197-2456(03)00112-0 15061154

[B19] Australian Government Department of Health and Ageing: Therapeutic Goods Administrationhttp://www.tga.gov.au/docs/about/publications.htm

[B20] CFR - Code of Federal Regulations Title 21 http://www.accessdata.fda.gov/scripts/cdrh/cfdocs/cfcfr/cfrsearch.cfm?cfrpart=312

[B21] Gatt JM Korgaonkar MS Schofield PR Harris A Clark CR Oakley KL Ram K Michaelson H Yap S Stanners M Wise V Williams LM The TWIN-E project in emotional wellbeing: study protocol and preliminary heritability results across four MRI and DTI measures Twin Res Hum Genet 2012 15 419 441 10.1017/thg.2012.12 22856376

[B22] Korgaonkar MS Grieve SM Etkin A Koslow SH Williams LM Using standardized fMRI protocols to identify patterns of prefrontal circuit dysregulation that are common and specific to cognitive and emotional tasks in major depressive disorder: first wave results from the iSPOT-D study Neuropsychopharmacology 2013 38 863 871 10.1038/npp.2012.252 23303059 PMC3671994

[B23] Liddell BJ Williams LM Rathjen J Shevrin H Gordon E A temporal dissociation of subliminal versus supraliminal fear perception: an event-related potential study J Cogn Neurosci 2004 16 479 486 10.1162/089892904322926809 15072682

[B24] Williams LM Palmer D Liddell BJ Song L Gordon E The ‘when’ and ‘where’ of perceiving signals of threat versus non-threat Neuroimage 2006 31 458 467 10.1016/j.neuroimage.2005.12.009 16460966

[B25] Gur RC Sara R Hagendoorn M Marom O Hughett P Macy L Turner T Bajcsy R Posner A Gur RE A method for obtaining 3-dimensional facial expressions and its standardization for use in neurocognitive studies J Neurosci Methods 2002 115 137 143 11992665 10.1016/s0165-0270(02)00006-7

[B26] Smith SM Jenkinson M Woolrich MW Beckmann CF Behrens TEJ Johansen-Berg H Bannister PR De Luca M Drobnjak I Flitney DE Niazy RK Saunders J Vickers J Zhang Y De Stefano N Brady JM Matthews PM Advances in functional and structural MR image analysis and implementation as FSL Neuroimage 2004 23 Suppl 1 S208 S219 15501092 10.1016/j.neuroimage.2004.07.051

[B27] Fischl B FreeSurfer Neuroimage 2012 62 774 781 10.1016/j.neuroimage.2012.01.021 22248573 PMC3685476

[B28] Frackowiak RSJ Ashburner JT Penny WD Zeki S Human Brain Function 2004 2 Waltham, MA: Academic

[B29] Grieve SM Clark CR Williams LM Peduto AJ Gordon E Preservation of limbic and paralimbic structures in aging Hum Brain Mapp 2005 25 391 401 10.1002/hbm.20115 15852381 PMC6871717

[B30] Grieve SM Williams LM Paul RH Clark CR Gordon E Cognitive aging, executive function, and fractional anisotropy: a diffusion tensor MR imaging study AJNR Am J Neuroradiol 2007 28 226 235 17296985 PMC7977408

[B31] Paul R Henry L Grieve SM Guilmette TJ Niaura R Bryant R Bruce S Williams LM Richard CC Cohen RA Gordon E The relationship between early life stress and microstructural integrity of the corpus callosum in a non-clinical population Neuropsychiatr Dis Treat 2008 4 193 201 18728817 10.2147/ndt.s1549 PMC2515911

[B32] Seckfort D Paul R Grieve S Vandenberg B Bryant R Williams L Clark C Cohen R Bruce S Gordon E Early life stress on brain structure and function across the lifespan: a preliminary study Brain Imaging Behav 2008 2 49 58 10.1007/s11682-007-9015-y

[B33] Grieve SM Korgaonkar MS Clark CR Williams LM Regional heterogeneity in limbic maturational changes: evidence from integrating cortical thickness, volumetric and diffusion tensor imaging measures Neuroimage 2011 55 868 879 10.1016/j.neuroimage.2010.12.087 21224000

[B34] Fischl B Sereno MI Tootell RB Dale AM High-resolution intersubject averaging and a coordinate system for the cortical surface Hum Brain Mapp 1999 8 272 284 10.1002/(SICI)1097-0193(1999)8:4<272::AID-HBM10>3.0.CO;2-4 10619420 PMC6873338

[B35] Fischl B Sereno MI Dale AM Cortical surface-based analysis. II: inflation, flattening, and a surface-based coordinate system Neuroimage 1999 9 195 207 10.1006/nimg.1998.0396 9931269

[B36] Dale AM Fischl B Sereno MI Cortical surface-based analysis. I. Segmentation and surface reconstruction Neuroimage 1999 9 179 194 10.1006/nimg.1998.0395 9931268

[B37] Dale AM Sereno MI Improved localization of cortical activity by combining Eeg and Meg with Mri Cortical surface reconstruction - a linear-approach J Cogn Neurosci 1993 5 162 176 10.1162/jocn.1993.5.2.162 23972151

[B38] Fischl B Dale AM Measuring the thickness of the human cerebral cortex from magnetic resonance images Proc Natl Acad Sci U S A 2000 97 11050 11055 10.1073/pnas.200033797 10984517 PMC27146

[B39] Fischl B Liu A Dale AM Automated manifold surgery: constructing geometrically accurate and topologically correct models of the human cerebral cortex IEEE Trans Med Imaging 2001 20 70 80 10.1109/42.906426 11293693

[B40] Fischl B Salat DH Busa E Albert M Dieterich M Haselgrove C van der Kouwe A Killiany R Kennedy D Klaveness S Montillo A Makris N Rosen B Dale AM Whole brain segmentation: automated labeling of neuroanatomical structures in the human brain Neuron 2002 33 341 355 10.1016/S0896-6273(02)00569-X 11832223

[B41] Fischl B Salat DH van der Kouwe AJ Makris N Segonne F Quinn BT Dale AM Sequence-independent segmentation of magnetic resonance images Neuroimage 2004 23 Suppl 1 S69 S84 15501102 10.1016/j.neuroimage.2004.07.016

[B42] Han X Jovicich J Salat D van der Kouwe A Quinn B Czanner S Busa E Pacheco J Albert M Killiany R Maguire P Rosas D Makris N Dale A Dickerson B Fischl B Reliability of MRI-derived measurements of human cerebral cortical thickness: the effects of field strength, scanner upgrade and manufacturer Neuroimage 2006 32 180 194 10.1016/j.neuroimage.2006.02.051 16651008

[B43] Jovicich J Czanner S Greve D Haley E van der Kouwe A Gollub R Kennedy D Schmitt F Brown G Macfall J Fischl B Dale A Reliability in multi-site structural MRI studies: effects of gradient non-linearity correction on phantom and human data Neuroimage 2006 30 436 443 10.1016/j.neuroimage.2005.09.046 16300968

[B44] Segonne F Dale AM Busa E Glessner M Salat D Hahn HK Fischl B A hybrid approach to the skull stripping problem in MRI Neuroimage 2004 22 1060 1075 10.1016/j.neuroimage.2004.03.032 15219578

[B45] Fischl B van der Kouwe A Destrieux C Halgren E Ségonne F Salat DH Busa E Seidman LJ Goldstein J Kennedy D Caviness V Makris N Rosen B Dale AM Automatically parcellating the human cerebral cortex Cereb Cortex 2004 14 11 22 10.1093/cercor/bhg087 14654453

[B46] Korgaonkar MS Grieve SM Koslow SH Gabrieli JD Gordon E Williams LM Loss of white matter integrity in major depressive disorder: evidence using tract-based spatial statistical analysis of diffusion tensor imaging Hum Brain Mapp 2011 32 2161 2171 10.1002/hbm.21178 21170955 PMC6870076

[B47] Behrens TE Johansen-Berg H Woolrich MW Smith SM Wheeler-Kingshott CM Boulby P Barker GJ Sillery EL Sheehan K Ciccarelli O Thompson AJ Brady JM Matthews PM Non-invasive mapping of connections between human thalamus and cortex using diffusion imaging Nat Neurosci 2003 6 750 757 10.1038/nn1075 12808459

[B48] Smith SM Jenkinson M Johansen-Berg H Rueckert D Nichols TE Mackay CE Watkins KE Ciccarelli O Cader MZ Matthews PM Behrens TE Tract-based spatial statistics: voxelwise analysis of multi-subject diffusion data Neuroimage 2006 31 1487 1505 10.1016/j.neuroimage.2006.02.024 16624579

[B49] Andersson JL Jenkinson M Smith S Non-linear Registration aka Spatial Normalisation. FMRIB Technical Report TR07JA2 2007 http://www.fmrib.ox.ac.uk/analysis/techrep

[B50] Andersson JL Jenkinson M Smith S Non-linear Optimisation. FMRIB Technical Report TR07JA1 2007 http://www.fmrib.ox.ac.uk/analysis/techrep

[B51] Mori S Oishi K Jiang H Jiang L Li X Akhter K Hua K Faria AV Mahmood A Woods R Toga AW Pike GB Neto PR Evans A Zhang J Huang H Miller MI van Zijl P Mazziotta J Stereotaxic white matter atlas based on diffusion tensor imaging in an ICBM template Neuroimage 2008 40 570 582 10.1016/j.neuroimage.2007.12.035 18255316 PMC2478641

[B52] Behrens TE Berg HJ Jbabdi S Rushworth MF Woolrich MW Probabilistic diffusion tractography with multiple fibre orientations: what can we gain? Neuroimage 2007 34 144 155 10.1016/j.neuroimage.2006.09.018 17070705 PMC7116582

[B53] Ja M Laurienti PJ Ra K Burdette JH An automated method for neuroanatomic and cytoarchitectonic atlas-based interrogation of fMRI data sets Neuroimage 2003 19 1233 1239 10.1016/S1053-8119(03)00169-1 12880848

[B54] Yarkoni T Poldrack RA Nichols TE Van Essen DC Wager TD Large-scale automated synthesis of human functional neuroimaging data Nat Methods 2011 8 665 670 10.1038/nmeth.1635 21706013 PMC3146590

[B55] Trivedi MH Rush AJ Wisniewski SR Nierenberg AA Warden D Ritz L Norquist G Howland RH Lebowitz B McGrath PJ Shores-Wilson K Biggs MM Balasubramani GK Fava M STAR*D Study Team Evaluation of outcomes with citalopram for depression using measurement-based care in STAR*D: implications for clinical practice Am J Psychiatry 2006 163 28 40 10.1176/appi.ajp.163.1.28 16390886

[B56] Korgaonkar MS Cooper NJ Williams LM Grieve SM Mapping inter-regional connectivity of the entire cortex to characterize major depressive disorder: a whole-brain diffusion tensor imaging tractography study Neuroreport 2012 23 566 571 10.1097/WNR.0b013e3283546264 22562047

[B57] He H Sui J Yu Q Turner J Ho B-C Sponheim SR Manoach DS Clark VP Calhoun VD Altered small-world brain networks in schizophrenia patients during working memory performance PLoS One 2012 7 e38195 10.1371/journal.pone.0038195 22701611 PMC3368895

[B58] Milo R Shen-Orr S Itzkovitz S Kashtan N Chklovskii D Alon U Network motifs: simple building blocks of complex networks Science 2002 298 824 827 10.1126/science.298.5594.824 12399590

[B59] Wang J-H Zuo X-N Gohel S Milham MP Biswal BB He Y Graph theoretical analysis of functional brain networks: test-retest evaluation on short- and long-term resting-state functional MRI data PLoS One 2011 6 e21976 10.1371/journal.pone.0021976 21818285 PMC3139595

[B60] Zhao X Liu Y Wang X Liu B Xi Q Guo Q Jiang H Jiang T Wang P Disrupted small-world brain networks in moderate Alzheimer’s disease: a resting-state FMRI study PLoS One 2012 7 e33540 10.1371/journal.pone.0033540 22457774 PMC3311642

[B61] Rosset A Spadola L Ratib O OsiriX: an open-source software for navigating in multidimensional DICOM images J Digit Imaging 2004 17 205 216 10.1007/s10278-004-1014-6 15534753 PMC3046608

[B62] Scheltens P Barkhof F Leys D Pruvo JP Nauta JJ Vermersch P Steinling M Valk J A semiquantative rating scale for the assessment of signal hyperintensities on magnetic resonance imaging J Neurol Sci 1993 114 7 12 10.1016/0022-510X(93)90041-V 8433101

[B63] Fair DA Schlaggar BL Cohen AL Miezin FM Dosenbach NU Wenger KK Fox MD Snyder AZ Raichle ME Petersen SE A method for using blocked and event-related fMRI data to study “resting state” functional connectivity Neuroimage 2007 35 396 405 10.1016/j.neuroimage.2006.11.051 17239622 PMC2563954

[B64] Gavrilescu M Stuart GW Rossell S Henshall K McKay C Sergejew AA Copolov D Egan GF Functional connectivity estimation in fMRI data: influence of preprocessing and time course selection Hum Brain Mapp 2008 29 1040 1052 10.1002/hbm.20446 17935181 PMC6871086

